# The Periodicity of Epidemics

**Published:** 1878-09

**Authors:** 


The Periodicity of Epidemics.—An interesting study of this subject, as applied to measles, in London, has been published by Dr. H. Courtenay Fox, in the *Medical Times and Gazette.* His conclusions are that a twofold law of periodicity has been disclosed. The successive epidemics have recurred with tolerable regularity, and without any marked exception to the rule, at intervals of about two years. Beside this, the severity of the disease appears to be subject to fluctuation, in accordance with a wider cycle of nine or ten years. If, in the future, measles observes the same rules by which its known course has been hitherto directed, we may assume that London is near the commencement of a fifth group of years, in which the epidemics will be rather more frequent and fatal than during the last decade.—*Medical and Surgical Reporter.*

